# Introduced bullfrogs are associated with increased *Batrachochytrium dendrobatidis* prevalence and reduced occurrence of Korean treefrogs

**DOI:** 10.1371/journal.pone.0177860

**Published:** 2017-05-31

**Authors:** Amaël Borzée, Tiffany A. Kosch, Miyeon Kim, Yikweon Jang

**Affiliations:** 1 Laboratory of Behavioral Ecology and Evolution, School of Biological Sciences, Seoul National University, Seoul, Republic of Korea; 2 One Health Research Group, College of Public Health, Medical and Veterinary Sciences, James Cook University, Townsville, Queensland, Australia; 3 Division of EcoScience, Ewha Womans University, Seoul, Republic of Korea; National Zoological Park, UNITED STATES

## Abstract

Bullfrogs, *Lithobates catesbeianus*, have been described as major vectors of the amphibian chytrid fungus, *Batrachochytrium dendrobatidis* (*Bd*). *Bd* is widespread throughout the range of amphibians yet varies considerably within and among populations in prevalence and host impact. In our study, the presence of *L*. *catesbeianus* is correlated with a 2.5 increase in *Bd* prevalence in treefrogs, and the endangered *Dryophytes suweonensis* displays a significantly higher *Bd* prevalence than the more abundant *D*. *japonicus* for the 37 sites surveyed. In addition, the occurrence of *L*. *catesbeianus* was significantly correlated with a decrease in presence of *D*. *suweonensis* at sites. We could not determine if it is the presence of bullfrogs as competitors or predators that is limiting the distribution of *D*. *suweonensis* or whether this is caused by bullfrogs acting as a reservoir for *Bd*. However, *L*. *catesbeianus* can now be added to the list of factors responsible for the decline of *D*. *suweonensis* populations.

## Introduction

A third of all amphibian species are under threat of extinction and more than two hundred are specifically under threats because of enigmatic diseases and threats [1, 2]. Evidence points to the chytrid fungus, *Batrachochytrium dendrobatidis* (*Bd*), as one of the major causes for population declines and extinction events [1–3]. However, *Bd* distribution is patchy, and its prevalence varies considerably with species susceptibility [4], latitude [5], temperature [6], and seasonality [7, 8]. For instance, colder months are associated with higher prevalence [9, 10] and mortality [11, 12]. The average rainfall and humidity at a site are also of major importance, because *Bd* is dispersed by waterborne zoospores [6, 13], and because all life stages of the fungus are sensitive to desiccation [14].

*Bd* occurs on all continents where amphibians are present [15] and the number of Asian countries where *Bd* has been detected is rapidly expanding [16–20]. The high diversity of endemic *Bd* genotypes in Korea suggests that *Bd* may have been present long before it was first detected [16, 21, 22]. However, it is likely that new genotype are being continually introduced through the pet trade of White’s Treefrogs, *Litoria caerulea* [23]. Currently, both the lethal panzootic *Bd* lineage “GPL” [24] and endemic genotypes are present in Korea [21].

American Bullfrogs (*Lithobates catesbeianus*) are known to be reservoirs for *Bd* [25–27], thus multiplying their negative impact on local amphibian species, originally limited to predation [28]. It has been hypothesized that this species is responsible for the dispersal of the hypervirulent *Bd*-GPL lineage globally [29]. Several factors make American Bullfrogs ideal Bd dispersers: (1) this species has exported globally and is raised for food in many countries [30], (2) American Bullfrogs do not develop clinical chytridiomycosis, even at high infection intensities [31], and (3) American Bullfrog populations tend to have high rates of infection [30].

Bullfrogs were first imported to the Republic of Korea in 1959 and then in larger numbers in the early 70’s. It took about 20 years for escaped individuals to establish wild populations. Currently, bullfrogs are listed as an invasive species in Korea. Although management policies were established in the past, these are no longer implemented; reviewed in [32].

Two treefrog species occur on the Korean peninsula: the endangered *Dryophytes suweonensis* (Suweon Treefrog, previously allocated to the *Hyla* genus [33]) and the widespread *D*. *japonicus* (previously *H*. *japonica*). Pending further taxonomic work, *D*. *suweonensis* is potentially synonymous to *D*. *immaculatus* (previously *H*. *immaculata* [34]) and *D*. *japonicus*, synonymous to *D*. *ussuriensis* [35, 36]. *Dryophytes suweonensis* is restricted to the western Korean lowlands and bisected by metropolitan Seoul [37, 38]. By contrast, *D*. *japonicus* ranges from Japan to Manchuria and from the Baikal Lakes to central China [39]. The distribution of *D*. *suweonensis* in completely within the distribution of *D*. *japonicus*. *Lithobates catesbeianus* is found throughout the range of both Hylid species in Korea. *Bd* prevalence has been previously reported in *D*. *japonicus* (53 out of 406 samples [21], 9 out of 10 samples [40]), but never in *D*. *suweonensis* (3 samples all tested negative for *Bd* [21]). However, *Bd* has been detected in *D*. *immaculatus* in China (1 out of 5 samples [18]).

The aim of this study was (1) to investigate *Bd* prevalence in the two Korean Hylid species, and (2) to study the effect of *L*. *catesbeianus* on the prevalence of *Bd* in these two treefrog species. We hypothesized that Bd and the presence of Bullfrogs has a negative effect on the occurrence of D. suweonensis. Due to its restricted range and population decline [38], the endangered *D*. *suweonensis* has low genetic diversity [41], and consequently a decreased ability to respond to *Bd* infection [42, 43], compared to the widespread and expanding *D*. *japonicus* [37]. Thus, we tested the prediction that *Bd* prevalence is higher in *D*. *suweonensis* than in *D*. *japonicus*. Findings of this study have important implications for the conservation of *D*. *suweonensis* as they highlight the threats posed by both American Bullfrogs and *Bd*.

## Material and methods

Our study was comprised two different phases, with separate field work and methodologies. We first studied *Bd* prevalence for *Dryophytes japonicus* and *D*. *suweonensis* at 37 sites. Secondly, we assessed the relationship between *Bd* infection and the presence of *L*. *catesbeianus* through aural surveys at 179 sites, including the 37 sites from the first phase of the study. The observations in this study were approved by the Ministry of Environment of the Republic of Korea (permits numbers 2015–03, 2015–05, 2015–6 and 2015–28), and thus qualifying for ethical assessment for experiments conducted on the endangered *D*. *suweonensis*. The experiment was also approved by the Animal Ethics Comity from Ewha Woman’s University (#2014–111).

### *Bd* sampling

**Fig 1:**
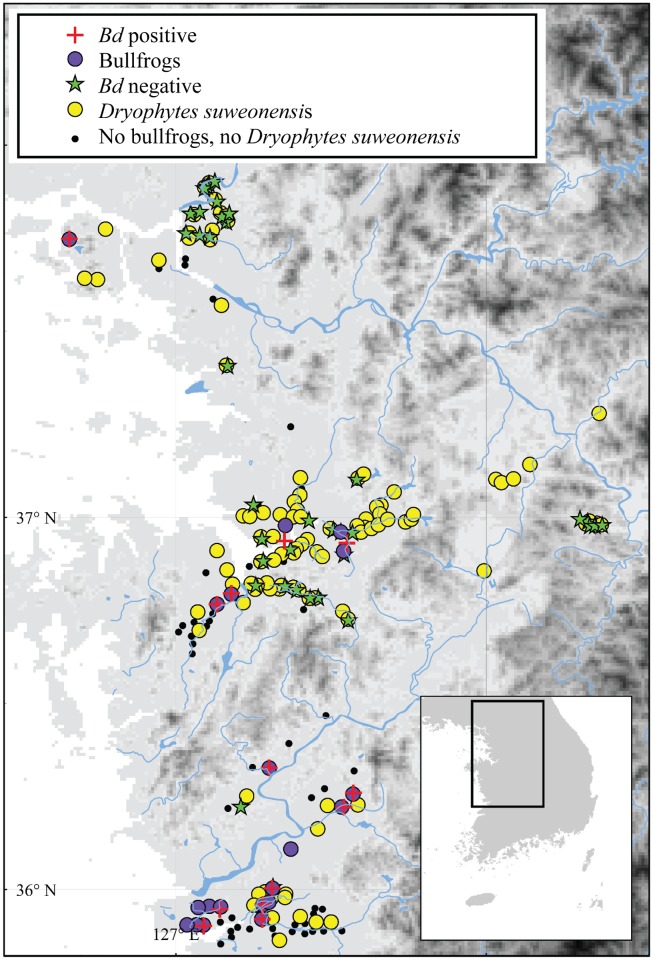
Summary map of sites sampled for both parts of this study. Description of the range of *Dryophytes suweonensis*, sites where the species was detected, sites where *Lithobates catesbeianus* was detected and sites where sampling for *Bd* sampling was conducted. This map was generated with ArcMap 9.3 (Environmental Systems Resource Institute, Redlands, California, USA; http://www.esri.com/).

Field sites were selected from previous studies on the presence of *D*. *suweonensis* [37, 38]. Site selection and sample number of *D*. *suweonensis* were restricted by permits given by the Ministry of Environment of the Republic of Korea, to 88 individuals from 37 sites, covering the totality of *D*. *suweonensis* range (Fig 1). A maximum of 20% of the population size at a given site, surveyed in 2014, was allowed for sampling (Table 1). Sampling for *D*. *japonicus* targeted all individuals encountered within the same rice paddies as the *D*. *suweonensis* caught (Fig 1). *Bd* sampling was performed between 15 May and 28 June, 2015, during the peak breeding seasons of these two species ([44]; S1 Table).

**Table 1:** Sampling sites and occurrences of frogs for the *Bd* surveys. Sampling for *Bd* infection was conducted at 37 sites throughout the range of *Dryophytes suweonensis* in Republic of Korea. The unit used for “Bullfrog” is the binary encoded prevalence, and the number of individuals for “*Ds*” and “*Dj*”.

Site	Season	Lat (°N)	Lon (°E)	Temp 1 d	Rain 1 d	Temp 3 mo	Rain 3 mo	Bullfrog	*Ds*	*Dj*	Prev *Ds*	Prev *Dj*
1	20	37.409	126.806	17.0	0.0	10.6	1.0	0	2	5	1	0
2	28	37.036	126.875	21.8	0.0	21.9	1.5	0	2	5	1	1
3	37	36.993	127.024	24.5	0.0	14.7	1.5	0	4	10	1	0
4	23	37.103	127.152	22.5	0.0	17.9	2.3	0	2	6	1	1
5	45	37.753	126.758	22.2	0.0	15.3	1.4	0	2	2	1	0
6	45	37.795	126.796	22.2	0.0	15.3	1.4	0	1	4	1	1
7	48	37.809	126.789	22.2	0.0	15.6	1.6	0	1	4	0	0
8	48	37.817	126.709	22.2	0.0	15.6	1.6	0	1	5	0	0
9	49	37.898	126.756	22.8	0.0	15.8	1.6	0	2	10	1	1
10	49	37.891	126.751	22.8	0.0	15.8	1.6	0	1	4	1	0
11	50	37.887	126.743	23.4	0.0	16.1	1.6	0	1	2	0	0
12	50	37.764	126.689	23.4	0.0	16.1	1.6	0	2	5	2	0
13	50	36.901	126.939	22.9	3.5	17.3	1.8	0	2	1	2	1
14	50	36.822	126.870	22.9	3.5	17.3	1.8	1	2	2	2	1
15	52	36.962	127.110	20.8	33.5	17.6	2.3	1	2	5	2	4
16	52	36.967	127.093	20.8	33.5	17.6	2.3	1	1	2	1	2
17	52	36.899	127.118	20.8	33.5	17.6	2.3	1	2	4	1	1
18	53	36.991	127.774	23.7	0.0	19.1	2.4	0	1	7	0	1
19	53	36.977	127.784	23.7	0.0	19.1	2.4	0	1	2	0	0
20	54	36.981	127.791	24.7	0.0	19.3	2.4	0	1	1	1	0
21	54	36.786	127.047	23.6	0.0	17.9	2.3	0	3	11	2	1
22	54	36.785	127.024	23.6	0.0	17.9	2.3	0	1	3	0	0
23	54	36.808	126.995	23.6	0.0	17.9	2.3	0	1	2	1	0
24	54	36.818	126.955	23.6	0.0	17.9	2.3	0	2	4	1	1
25	10	37.818	126.810	18.1	0.0	8.3	1.3	0	3	1	2	1
26	19	36.725	127.129	19.5	0.0	17.3	1.8	0	3	6	1	1
27	24	36.944	126.898	22.4	0.0	18.1	2.3	1	3	6	2	4
28	27	37.760	126.731	18.6	0.0	11.4	1.1	0	3	6	3	0
29	29	37.850	126.777	21.6	0.0	11.8	1.1	0	5	6	1	1
30	30	36.916	126.975	22.1	0.0	13.4	1.5	1	1	4	1	2
31	31	36.224	126.841	18.8	4.5	7.5	1.6	0	1	6	0	0
32	32	36.009	126.927	20.0	0.0	15.5	2.3	1	10	4	9	2
33	33	37.904	126.771	20.2	0.0	12.7	1.1	0	7	17	3	0
34	35	36.981	127.811	23.0	0.0	16.0	1.9	0	5	2	3	0
35	39	36.960	127.141	25.3	0.0	15.4	1.5	1	5	10	2	8
36	40	36.997	127.751	23.9	1.0	17.6	2.0	0	3	2	1	0
37	41	37.823	126.731	22.5	0.0	14.6	1.4	0	2	7	1	0

Lat. stands for latitude and Lon. for longitude. Temp. stands for temperature. Ds is *D*. *suweonensis*, Dj is *D*. *japonicus* and Prev. stands for *Bd* prevalence. M for male and F for female. Season is the number of days since the first calls were heard for the two species in 2015. Temperature is in °C and precipitation in mm.

To detect *Bd* infection, individuals were first captured by rapidly placing a hand on the individual, covered with a single-use vinyl bag (Clean Wrap, # 365780167; Gimhae, Republic of Korea), then kept in separate bags to prevent cross-contamination and finally swabbed on the epidermis. Sampling was conducted at the breeding sites, between 4 pm and 4 am. Swabbing was done with sterile fine-tip swabs (Medical Wire & Equipment Co Ltd; Corsham, UK) wearing a new pair of vinyl gloves for each frog. Frogs were systematically swabbed five times on each toe of the hind legs, each foot, the inner thighs, and both sides of the abdomen. Since the two Hylid species are difficult to distinguish morphologically [45], buccal swabs from each individual were collected for species identification through mtDNA CO1 sequencing, following the protocol developed by Jang [46]. Swabs were stored in plastic tubes at -20°C until DNA extraction. All individuals were released at the point of capture as soon as the site was sampled, *i*.*e*. within 2 h of capture.

### Positive control for *Bd* detection

Since bullfrogs are a known reservoir for *Bd* in the Republic of Korea we first swabbed 7 individuals in order to obtain a positive control for PCR. The bullfrogs were obtained from irrigating ditches between rice-paddies in Hwaseong (37.155977°N, 126.721290°E), caught with butterfly types of nets, adequately sterilized and dried between sites, and swabbed following the *Bd* swabbing protocol described above. None of the individual caught displayed any sign of clinical disease or abnormality.

We obtained two positive reactions from two different individuals out of the seven *L*. *catesbeianus* swabbed following the protocol described below. The samples were then sent to Macrogen Inc. (Seoul, Republic of Korea) for direct sequencing in the forward direction on an ABI PRISM 3100 automatic sequencer (Applied Biosystems Inc.; USA). The sequences were manually trimmed in Geneious (v9.04, Biomatters Limited, Auckland, New Zealand) and blasted on the Basic Local Alignment Search Tool from the NCBI portal (http://blast.ncbi.nlm.nih.gov/Blast.cgi). One of our sequences was 100% identical to *Batrachochytrium dendrobatidis* accession number JX983045.1, collected from a *L*. *catesbeianus* [21]. This sample was used as positive control for all PCRs in our study, and maintained at -20°C in 1.5 mL tubes.

### DNA extraction and *Bd* detection

DNA was extracted following a modified version of the four-step protocol developed by Hyatt [47]. The protocol consisted in incubating each swab in 50 μL of Prep Man Ultra (Applied Biosystems; California, USA) at 100°C for 10 min, before cooling at room temperature for 2 min, centrifugation at 13,000 rpm for 3 min, and transfer of the supernatant. Each sample was subsequently diluted 1:5 with PCR grade water before PCR. We used a nested PCR developed by Goka [48] for *Bd* detection. This reaction consisted of two PCRs that were the same except for the primers. The PCR1 used the primers Bd18SF1 (5’-TTTGTACACACCGCCCGTCGC-3’) and Bd28SR1 (5’-ATATGCTTAAGTTCAGCGGG-3’) from Goka [48] while the PCR2 used the primers Bd1a (5’-CAGTGTGCCATATGTCACG-3’) and Bd2a (5’-CATGGTTCATATCTGTCCAG-3’) from Annis [49]. Each PCR contained 12.8 μL of distilled water, 2.0 μL of (10x) buffer, 1.2 μL of dNTPs (final concentration of 0.06 mM), 0.4 μL of each primer, 2 μL of BSA (4000 ng/ μL), 0.2 μL of Takara Taq polymerase and 1 μL of DNA. The thermocycler (PTC-100, BIO-RAD; California; USA) was programmed at 94°C for 3 min, followed by 35 cycles at 94°C for 30 sec, 50°C for 30 s and 72°C for 2 min, with a terminal elongation at 72°C for 7 min. Samples were then run on a 1.5% agarose gel during a 15 min electrophoresis, and pre-stained with MaestroSafe dye (Maestrogen; Las Vegas, Nevada, USA).

All samples in this study were considered positive if a band was visible at the same size as the positive control (at approximatively 300 bp). If any of the negative controls displayed a band, all PCR results from the run were discarded and all reactions ran again.

Each treefrog sample was run in two independent PCR replicates along with positive (known *Bd* sample) and negative controls (PCR master-mix and distilled water). A sample was considered positive if both replicates were positive. Two samples for *D*. *suweonensis* and one for *D*. *japonicus* (all males, respectively from localities 15, 20 and 29; Table 1) were positive for one of the replicates only and were consequently run a third time. All three samples were positive for the third replicate and thus considered positive.

### Environmental variables and *Bd* infection

To assess the impact of the environment on *Bd* prevalence, we collected the average temperature (°C) and precipitation (mm) for the 3 months prior to sampling at each site (Table 2). The data were collected from the closest weather stations of the National Weather Service Stations of Korea (http://www.kma.go.kr/weather/observation/aws_table_popup.jsp). Weather data on the days of sampling were not included in the analysis due to a high correlation with the 3-month average (Pearson correlation; *r* = 0.84; *P* < 0.001). The other variables mentioned.

**Table 2:** Univariate GLM with *Bd* infection as the dependent variable to assess the effect of the 6 variables collected on the *Bd* infection status. Temp. (3 mo) and Rain (3 mo) denote temperature and precipitation for three months prior to sampling.

	Mean Square	*df*	*F*	*P*-value
Bullfrogs	8.29	1	55.16	< 0.001
Species	6.87	1	45.74	< 0.001
Sex	0.04	1	0.26	0.611
Season	0.13	1	0.88	0.349
Temp. (3 mo)	0.08	1	0.52	0.473
Rain (3 mo)	0.01	1	0.09	0.763
Error	39.65	264		

### Aural surveys

We conducted presence/absence transect surveys to examine the impact of *L*. *catesbeianus* on the distribution of *D*. *suweonensis* for the entire reported range of *D*. *suweonensis* (Borzée et al., in prep). *Dryophytes japonicus* was not included in this analysis due to its ubiquitous presence at all sites studied.

We used preliminary data on the distribution of *D*. *suweonensis* collected from 2013 and 2014 to select sites that fulfilled the ecological requirements for this species throughout its whole range (see [37]). This resulted in the selection of 179 sites with a high probability of *D*. *suweonensis* occurrence. All sites selected were below 35 m a.s.l. and located in large plains managed for rice cultivation. Another common landscape feature for the occurrence of *D*. *suweonensis* among sites was the relatively low abundance of man-made structures (e.g. houses, factories, greenhouses). Auditory surveys were conducted for both *D*. *suweonensis* and *L*. *catesbeianus*, using the method described by Borzée and Jang [38]. Surveys were conducted during the weeks of highest calling activity, between 15 May and 21 June 2015, and during the time of day when the two species are known to be active, between 6 pm and midnight [37, 38].

Each survey was 10 min long and conducted while walking along the longest straight line available in rice paddies. Transect selection was facilitated by the grid structure of modern paddy fields. We tested whether 10 min transects accurately estimate the presence/absence of *D*. *suweonensis* through 3 replicate samplings at 10 sites. No significant variations in occurrence were found between replicates (repeated ANOVA; χ = 0.33; *df* = 2, *P* = 0.630), indicating that this method is effective at estimating the presence of *D*. *suweonensis* at a site.

### Statistical analysis

Multicollinearity among all variables was examined prior to the statistical analysis through the use of Variance Inflation Factors (VIF [50]), instead of bivariate correlations to avoid simple pairwise comparisons of correlations [51]. Because VIF values were between 1.003 and 2.340, all variables were included in the subsequent analyses. To assess the factors important for *Bd* prevalence, we ran a univariate General Linear Model (GLM), with *Bd* infection as a dependent variable. The fixed factors were bullfrog presence, species, and sex; and the covariates were season, temperature, and precipitation for the three months prior to sampling. Species, sex, and *Bd* occurrence were binary encoded. Species was either *D*. *suweonensis* or *D*. *japonicus*. Season was defined as the number of days after 5 May 2015, the first day for both treefrog species to produce advertisement calls in 2015. We then ran a pair of ANOVAs in order to determine statistical significance for variations in *Bd* infection between (1) sites with and without bullfrogs, and (2) the two Hylid species. Prior to the analysis the homogeneity of variance was tested through Levene’s test (*P* > 0.016), which was subsequently ignored as marginally influential to the data analysis. The data presented relies on the calculation of averages and frequencies to indicate the directionalities in prevalence. Finally, we assessed the impact of *L*. *catesbeianus* on the distribution of *D*. *suweonensis* through Fisher’s exact test. All analyses were conducted with SPSS (v. 21.0, SPSS, Inc., Chicago, IL, USA).

## Results

### *Bd* prevalence

*Bd* was detected in both Hylid species, at 31 of 37 sites (83.78%). *Lithobates catesbeianus* were present at eight sites, while both male *Dryophytes suweonensis* (*n* = 70) and *D*. *japonicus* (*n* = 151) were present at all 37 sites. However, female *D*. *suweonensis* (*n* = 19) were only present at 11 sites, and female *D*. *japonicus* (*n* = 31) at 16 sites (Appendix 1).

The results of the GLM indicated that only two variables, bullfrog presence/absence and species, were significant predictors of *Bd* infection (*F* ≥ 45.74; *P* < 0.001; Table 2). In contrast, sex, season, temperature, and precipitation (averaged for the 3 months prior to sampling), were not significant in our model.

**Fig 2:**
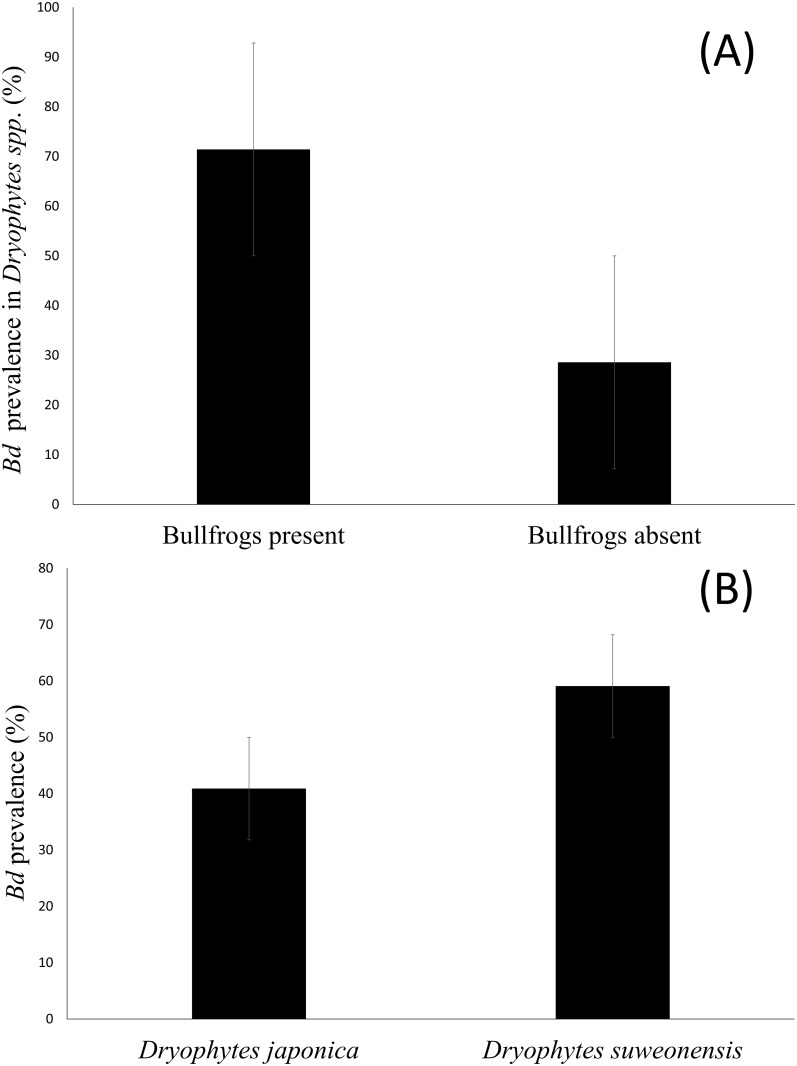
(A) Percentage of Korean treefrogs infected by *Bd* as a function of bullfrog presence. The graph is representative of the whole treefrog population for both species at sites with bullfrogs and at sites without bullfrogs. (B) *Bd* prevalence for each treefrog species in percent. The *Bd* prevalence was lower in *Dryophytes japonicus* by a factor of 0.7. For both figures, the vertical thin bars at the center of the percentage thick bars are standard error bars.

For both treefrog species combined, *Bd* infection was more likely in populations with bullfrogs (71.4%, *n* = 45), than the converse (28.6%, *n* = 18; ANOVA; Mean square = 10.14; *df* = 1, 269; *F* = 71.34; *P* < 0.001; Fig 2A). When considering the sites with *D*. *japonicus* regardless of the presence of *D*. *suweonensis*, 24 out of 37 frogs (35.1%) were infected with *Bd* in the presence of bullfrogs, while 12 out of 145 frogs (8.3%) only were infected with *Bd* in the absence of bullfrogs. When sites with *D*. *suweonensis* were considered regardless of the presence of *D*. *japonicus*, 21 out of 26 frogs (80.8%) were infected with *Bd* in the presence of bullfrogs, while only for 31 out of 63 frogs (49.2%) were infected with *Bd* in the absence of bullfrogs. When considering all treefrogs, *Bd* prevalence in *D*. *japonicus* (40.9%; *n* = 36) was significantly lower (ANOVA; Mean square = 8.98, *df* = 1,269; *F* = 47.56; *P* < 0.001) than in *D*. *suweonensis* (59.1%; *n* = 52; Fig 2B).

### *Dryophytes suweonensis* distribution

In addition to studying the impact of *Bd* across a subset of *D*. *suweonensis* populations, we also investigated how the presence of bullfrogs influenced the occurrence of *D*. *suweonensis* at 179 sites (Fig 1). The results of our field surveys showed that *D*. *suweonensis* was present at 114 sites and *L*. *catesbeianus* at 22 sites. *Dryophytes suweonensis* was only present at 9 of the 22 sites with *L*. *catesbeianus* (Table 3). The presence of *D*. *suweonensis* was significantly negatively related with the occurrence of *L*. *catesbeianus* (Fisher’s exact test; *χ*^*2*^ = 5.31, *df* = 1, *P* = 0.031; *Phi* = -0.17, *P* = 0.018). This finding indicates that *D*. *suweonensis* is less likely to occur at sites where *L*. *catesbeianus* is found.

**Table 3:** Contingency table for occurrences of *Dryophytes suweonensis* and *Lithobates catesbeianus* throughout the range of *D*. *suweonensis*. The expected counts are the value expected if there were no relationship between the occurrence of *D*. *suweonensis* and *L*. *catesbeianus*.

		*Dryophytes suweonensis*		Total
		Absent	Present	
*Lithobates catesbeianus*	Absent	52 (57.0)	105 (100.0)	157
	Present	13 (8.0)	9 (14.0)	22
	Total	65	114	179

The numbers in cells are the observed counts, and the numbers in parentheses are the expected counts.

## Discussion

We show that *Bd* prevalence in Korean treefrogs was highest at sites with *Lithobates catesbeianus*, suggesting that this species is a vector or reservoir of the fungal pathogen. We also found a negative correlation between the presence of bullfrogs and occurrence of *Dryophytes suweonensis*. Finally, we found that *Bd* prevalence was much higher for *D*. *suweonensis* than it was for *D*. *japonicus*. These findings suggest that either *Bd* infection or *L*. *catesbeianus*, or possibly both factors together, may play a role in population decline of *D*. *suweonensis*, thus hastening the possible loss of this species.

Although *Bd* prevalence was high in *D*. *suweonensis*, it is unknown if *Bd* directly causes declines in this species as mass mortality events such as those reported in other *Bd* infected species [2] were not observed in *D*. *suweonensis* during > 300 days of field work conducted between 2013 and 2015. It appears that *Bd* may not be causing declines in this species, although sub-lethal effects of *Bd* (e.g. decreased growth, [52, 53]) cannot be ruled out. It is possible that both treefrog species are tolerant to *Bd* infection. Further studies including *Bd* challenge experiments in the laboratory and long term monitoring in the field are necessary. Long term monitoring would also resolve potential false-negative *Bd* prevalence due to low sample sizes, which could have introduced bias if *Bd* had not been detected at more than the seven sites from this study.

One explanation for the difference in *Bd* prevalence between the two treefrog species is that *D*. *suweonensis* might be immunocompromised due to low genetic diversity. *Dryophytes suweonensis* is an endangered species with a restricted range and a low genetic diversity (Borzée et al., unpublished). Thus, we expected that the genetic bottleneck associated with this species would have had a negative impact on immunity, notably through a loss of variability in the Major Histocompatibility Complex (MHC), which has been associated with *Bd* resistance in other amphibian species (Bataille et al. 2015, Savage et al. 2011, 2016).

The variation in *Bd* prevalence between the two treefrog species may also be explained by the differences in habitat use by the two species. During the breeding season, male *D*. *suweonensis* produce advertisement calls at the center of flooded rice paddies, whereas male *D*. *japonicus* call from the banks of rice paddies, outside of water [54]. Such a niche segregation between the two species results in *D*. *suweonensis* being more closely associated with water than *D*. *japonicus*. Other studies have shown evidence of an association between increased *Bd* prevalence and association with water [55]. In addition, *D*. *suweonensis* hibernates on the bank of the rice paddies, while *D*. *japonicus* hibernates in forests (Borzée et al., unpublished). Differential uses of habitats between the two treefrog species can lead to a higher prevalence of *Bd* for *D*. *suweonensis* than for *D*. *japonicus*. Future studies should examine seasonal variations in *Bd* prevalence and intensity, through quantitative PCR analysis, potentially demonstrating a variation in mortality depending on seasons or during metamorphosis [56].

The difference in *Bd* prevalence between *D*. *japonicus* and *D*. *suweonensis* could also lie in behavioral differences. Some species have been shown to behaviorally reduce the chance of *Bd* infection by positively selecting *Bd*-free microhabitats [57], or displaying thermoregulatory behavior, such as basking, e.g. *Atelopus zeteki* [58]. *Dryophytes suweonensis* is active at higher temperatures than *D*. *japonicus* [54], but it is not known whether these two species differ in basking behavior. Thus, despite *D*. *suweonensis* being active at higher temperature, a more pronounced basking by *D*. *japonicus* could lead to the difference in *Bd* prevalence between the two species.

The negative impact of *L*. *catesbeianus* on *D*. *suweonensis* may result from a combination of several factors. *Lithobates catesbeianus* is a known vector of *Bd* [26, 59, 60], but it also preys on smaller species [61], using male calls as a detection beacon [62]. It has also been shown to predate on other Korean species, such as *Pelophylax chosenicus* [32, 63] and *D*. *japonicus* (A. Borzée personal observation, June 2015; 36.137594°N, 127.381514°E). Thus, *D*. *suweonensis* may be avoiding sites where *L*. *catesbeianus* occurs. Alternatively, it could also be possible for males to be quiet at sites where *L*. *catesbeianus* is present in order to avoid predation. This would result in a larger population of *D*. *suweonensis* than recorded, although it would not last longer than the generation length of the species as females would be less efficient at locating males for breeding.

Alternatively, bullfrogs have been shown to compete with other species for food resources, during both larval [64, 65] and adult stages [66]. *Lithobates catesbeianus* breeds in ditches and deeper aquatic environments than in rice paddies where *D*. *suweonensis* breeds. Due to this apparent segregation in habitat, exploitative competition based on resource exploitation between *D*. *suweonensis* and *L*. *catesbeianus* seems unlikely as the two species use different food resources and egg deposition sites.

Our research highlights that the endangered *D*. *suweonensis* is currently at risk of further population declines with the expansion of the invasive *L*. *catesbeianus*. It is of prime importance to prevent further expansion of the invasive *L*. *catesbeianus*, and to remove individuals already present. We recommend an increase in governmental policies that offer financial incentives for the capture of bullfrogs.

## Supporting information

S1 Table *Bd* prevalence for the two *Dryophytes* species. Sampling sites, sex of frogs and Bd prevalence for this study.
(DOCX)

S2 Table Call surveys for *Dryophytes suweonensis* and *Lithobates catesbeianus*. For “*L*. *catesbeianus*” and “*D*. *suweonensis*”, data is binary encoded: 0 = absent and 1 = present.
(DOCX)

## References

[1] Stuart SN, Chanson JS, Cox NA, Young BE, Rodrigues AS, Fischman DL, et al Status and trends of amphibian declines and extinctions worldwide. Science. 2004;306(5702): 1783–6. 10.1126/science.1103538 15486254

[2] Skerratt LF, Berger L, Speare R, Cashins S, McDonald KR, Phillott AD, et al Spread of chytridiomycosis has caused the rapid global decline and extinction of frogs. EcoHealth. 2007;4(2): 125–34.

[3] Lips KR, Brem F, Brenes R, Reeve JD, Alford RA, Voyles J, et al Emerging infectious disease and the loss of biodiversity in a Neotropical amphibian community. Proc Natl Acad Sci USA. 2006;103(9): 3165–70. 10.1073/pnas.0506889103 16481617 PMC1413869

[4] Woodhams DC, Alford RA. Ecology of chytridiomycosis in rainforest stream frog assemblages of tropical Queensland. Conserv Biol. 2005;19(5): 1449–59.

[5] Kriger KM, Pereoglou F, Hero J-m. Latitudinal variation in the prevalence and intensity of chytrid (*Batrachochytrium dendrobatidis*) infection in eastern Australia. Conserv Biol. 2007;21(5): 1280–90. 10.1111/j.1523-1739.2007.00777.x 17883493

[6] Longcore JE, Pessier AP, Nichols DK. *Batrachochytrium dendrobatidis gen*. et *sp*. *nov*., a chytrid pathogenic to amphibians. Mycologia. 1999;91(2): 219–27.

[7] Kriger KM, Hero JM. Large-scale seasonal variation in the prevalence and severity of chytridiomycosis. J Zool. 2007;271(3): 352–9.

[8] Whitfield SM, Kerby J, Gentry LR, Donnelly MA. Temporal variation in infection prevalence by the amphibian chytrid fungus in three species of frogs at La Selva, Costa Rica. Biotropica. 2012;44(6): 779–84.

[9] Berger L, Speare R, Hines H, Marantelli G, Hyatt A, McDonald K, et al Effect of season and temperature on mortality in amphibians due to chytridiomycosis. Aust Vet J. 2004;82(7): 82. 10.1111/j.1751-0813.2004.tb11137.x 15354853

[10] Ouellet M, Mikaelian I, Pauli BD, Rodrigue J, Green DM. Historical evidence of widespread chytrid infection in North American amphibian populations. Conserv Biol. 2005;19(5): 1431–40.

[11] Bradley GA, Rosen PC, Sredl MJ, Jones TR, Longcore JE. Chytridiomycosis in native Arizona frogs. J Wildl Dis. 2002;38(1): 206–12. 10.7589/0090-3558-38.1.206 11838218

[12] Savage AE, Zamudio KR. MHC genotypes associate with resistance to a frog-killing fungus. Proc Natl Acad Sci USA. 2011;108(40): 16705–10. 10.1073/pnas.1106893108 21949385 PMC3189034

[13] Piotrowski JS, Annis SL, Longcore JE. Physiology of *Batrachochytrium dendrobatidis*, a chytrid pathogen of amphibians. Mycologia. 2004;96(1): 9–15. 21148822

[14] Johnson M, Berger L, Phillips L, Speare R. Fungicidal effects of chemical disinfectants, UV light, desiccation and heat on the amphibian chytrid, *Batrachochytrium dendrobatidis*. Dis Aquat Organ. 2003;57: 255–60. 10.3354/dao057255 14960039

[15] Murray KA, Skerratt LF, Speare R, McCallum H. Impact and Dynamics of Disease in Species Threatened by the Amphibian Chytrid Fungus, *Batrachochytrium dendrobatidis*. Conserv Biol. 2009;23(5): 1242–52. 19774709 10.1111/j.1523-1739.2009.01211.x

[16] Swei A, Rowley JJ, Rödder D, Diesmos ML, Diesmos AC, Briggs CJ, et al Is chytridiomycosis an emerging infectious disease in Asia? PloS one. 2011;6(8): e23179 10.1371/journal.pone.0023179 21887238 PMC3156717

[17] Erismis UC, Konuk M, Yoldas T, Agyar P, Yumuk D, Korcan SE. Survey of Turkey’s endemic amphibians for chytrid fungus *Batrachochytrium dendrobatidis*. Dis Aquat Organ. 2014;111(2): 153–7. 10.3354/dao02742 25266902

[18] Zhu W, Bai C, Wang S, Soto-Azat C, Li X, Liu X, et al Retrospective survey of museum specimens reveals historically widespread presence of Batrachochytrium dendrobatidis in China. Ecohealth. 2014;11(2): 241–50. 10.1007/s10393-013-0894-7 24419667

[19] Reshetnikov AN, Chestnut T, Brunner JL, Charles K, Nebergall EE, Olson DH. Detection of the emerging amphibian pathogens *Batrachochytrium dendrobatidis* and ranavirus in Russia. Dis Aquat Organ. 2014;110: 235–40. 10.3354/dao02757 25114047

[20] Molur S, Krutha K, Paingankar MS, Dahanukar N. Asian strain of *Batrachochytrium dendrobatidis* is widespread in the Western Ghats, India. Dis Aquat Organ. 2015;112: 251–5. 10.3354/dao02804 25590776

[21] Bataille A, Fong JJ, Cha M, Wogan GO, Baek HJ, Lee H, et al Genetic evidence for a high diversity and wide distribution of endemic strains of the pathogenic chytrid fungus *Batrachochytrium dendrobatidis* in wild Asian amphibians. Mol Ecol. 2013;22(16): 4196–209. 10.1111/mec.12385 23802586

[22] Moriguchi S, Tominaga A, Irwin KJ, Freake MJ, Suzuki K, Goka K. Predicting the potential distribution of the amphibian pathogen *Batrachochytrium dendrobatidis* in East and Southeast Asia. Dis Aquat Organ. 2015;113(3): 177–85. 10.3354/dao02838 25850395

[23] Yang H. Survey of amphibian chytrid fungus Batrachochytrium dendrobatidis in free-ranging and imported populations of amphibians in South Korea. Seoul, Korea: MSc Thesis, Seoul National University; 2010.

[24] Farrer RA, Weinert LA, Bielby J, Garner TW, Balloux F, Clare F, et al Multiple emergences of genetically diverse amphibian-infecting chytrids include a globalized hypervirulent recombinant lineage. Proc Natl Acad Sci. 2011;108(46): 18732–6. 10.1073/pnas.1111915108 22065772 PMC3219125

[25] Mazzoni R, Cunningham AA, Daszak P, Apolo A, Perdomo E, Speranza G. Emerging pathogen of wild amphibians in frogs (*Rana catesbeiana*) farmed for international trade. Emerg Infect Dis. 2003;9(8): 995 10.3201/eid0908.030030 12967500 PMC3020601

[26] Daszak P, Strieby A, Cunningham AA, Longcore J, Brown C, Porter D. Experimental evidence that the bullfrog (*Rana catesbeiana*) is a potential carrier of chytridiomycosis, an emerging fungal disease of amphibians. Herpetological Journal. 2004;14(4): 201–7.

[27] Miaud C, Dejean T, Savard K, Millery-Vigues A, Valentini A, Gaudin NCG, et al Invasive North American bullfrogs transmit lethal fungus *Batrachochytrium dendrobatidis* infections to native amphibian host species. Biol Invasions. 2016: 1–10.

[28] Leivas PT, Leivas FW, Moura MO. Diet and trophic niche of *Lithobates catesbeianus* (Amphibia: Anura). Zoologia (Curitiba). 2012;29(5): 405–12.

[29] Schloegel LM, Toledo LF, Longcore JE, Greenspan SE, Vieira CA, Lee M, et al Novel, panzootic and hybrid genotypes of amphibian chytridiomycosis associated with the bullfrog trade. Mol Ecol. 2012;21(21): 5162–77. 10.1111/j.1365-294X.2012.05710.x 22857789

[30] Garner TW, Perkins MW, Govindarajulu P, Seglie D, Walker S, Cunningham AA, et al The emerging amphibian pathogen *Batrachochytrium dendrobatidis* globally infects introduced populations of the North American bullfrog, *Rana catesbeiana*. Biol Lett. 2006;2(3): 455–9. 10.1098/rsbl.2006.0494 17148429 PMC1686185

[31] Hanselmann R, Rodrıguez A, Lampo M, Fajardo-Ramos L, Aguirre AA, Kilpatrick AM, et al Presence of an emerging pathogen of amphibians in introduced bullfrogs *Rana catesbeiana* in Venezuela. Biol Cons. 2004;120(1): 115–9.

[32] Park D, Min M-S, Lasater K, Song J-Y, Suh J-H, Son S-H, et al. Conservation of amphibians in South Korea: I. Das, M. Wilkinson, and H. Heatwole (eds.); 2014.

[33] Duellman WE, Marion AB, Hedges SB. Phylogenetics, classification, and biogeography of the treefrogs (Amphibia: Anura: Arboranae). Zootaxa. 2016;4104(1): 1–109. 10.11646/zootaxa.4104.1.1 27394762

[34] Li J-T, Wang J-S, Nian H-H, Litvinchuk SN, Wang J, Li Y, et al Amphibians crossing the bering land bridge: evidence from holarctic treefrogs (Hyla, Hylidae, Anura). Mol Phylogenet Evol. 2015;87: 80–90. 10.1016/j.ympev.2015.02.018 25765368

[35] Fei L, Ye C-Y, Huang Y-Z, Liu M. Atlas of amphibians of China: Henan Science and Technology Press; 1999.

[36] Dufresnes C, Litvinchuk SN, Borzée A, Jang Y, Li J-T, Miura I, et al Phylogeography reveals an ancient cryptic radiation in East-Asian tree frogs (*Hyla japonica* group) and complex relationships between continental and island lineages. BMC Evol Biol. 2016;16(253). 10.1186/s12862-016-0814-x PMC5121986 27884104

[37] Roh G, Borzée A, Jang Y. Spatiotemporal distributions and habitat characteristics of the endangered treefrog, *Hyla suweonensis*, in relation to sympatric *H*. *japonica*. Ecol Inform. 2014;24: 78–84.

[38] Borzée A, Jang Y. Description of a seminatural habitat of the endangered Suweon treefrog, *Hyla suweonensis*. Anim Cells Syst. 2015;19(3): 1–5.

[39] Kuzmin S, Maslova I, Matsui M, Liang F, Kaneko Y. Hyla japonica. The IUCN Red List of Threatened Species. 2016.

[40] Yang H, Baek H, Speare R, Webb R, Park S, Kim T, et al First detection of the amphibian chytrid fungus *Batrachochytrium dendrobatidis* in free-ranging populations of amphibians on mainland Asia: survey in South Korea. Dis Aquat Organ. 2009;86: 9–13. 10.3354/dao02098 19899344

[41] Chun S, Chung E, Voloshina I, Chong JR, Lee H, Min M-S. Genetic Diversity of Korean Tree Frog (*Hyla suweonensis* and *Hyla japonica*): Assessed by Mitochondrial Cytochrome b Gene and Cytochrome Oxidase Subunit I Gene. Kor J Herpetol. 2012;4: 31–41.

[42] O'brien S, Roelke M, Marker L, Newman A, Winkler C, Meltzer D, et al Genetic basis for species vulnerability in the cheetah. Science. 1985;227(4693): 1428–34. 2983425 10.1126/science.2983425

[43] Spielman D, Brook BW, Briscoe DA, Frankham R. Does inbreeding and loss of genetic diversity decrease disease resistance? Conserv Genet. 2004;5(4): 439–48.

[44] Yoo E, Jang Y. Abiotic effects on calling phenology of three frog species in Korea. Anim Cells Syst. 2012;16(3): 260–7.

[45] Borzée A, Park S, Kim A, Kim H-T, Jang Y. Morphometrics of two sympatric species of tree frogs in Korea: a morphological key for the critically endangered *Hyla suweonensis* in relation to *H*. *japonica*. Anim Cells Syst. 2013;17(5): 348–56.

[46] Jang Y, Hahm EH, Lee H-J, Park S, Won Y-J, Choe JC. Geographic variation in advertisement calls in a tree frog species: gene flow and selection hypotheses. PloS one. 2011;6(8): e23297 10.1371/journal.pone.0023297 21858061 PMC3157349

[47] Hyatt A, Boyle D, Olsen V, Boyle D, Berger L, Obendorf D, et al Diagnostic assays and sampling protocols for the detection of *Batrachochytrium dendrobatidis*. Dis Aquat Organ. 2007;73: 175–92. 10.3354/dao073175 17330737

[48] Goka K, Yokoyama J, Une Y, Kuroki T, Suzuki K, Nakahara M, et al Amphibian chytridiomycosis in Japan: distribution, haplotypes and possible route of entry into Japan. Mol Ecol. 2009;18(23): 4757–74. 10.1111/j.1365-294X.2009.04384.x 19840263

[49] Annis SL, Dastoor FP, Ziel H, Daszak P, Longcore JE. A DNA-based assay identifies *Batrachochytrium dendrobatidis* in amphibians. J Wildl Dis. 2004;40(3): 420–8. 10.7589/0090-3558-40.3.420 15465708

[50] Zuur A, Ieno E, Walker N, Saveliev A, Smith G. Mixed effects models and extensions in ecology with R. New York: Springer; 2009 574 p.

[51] Ernst R, Massemin D, Kowarik I. Non-invasive invaders from the Caribbean: the status of Johnstone’s Whistling frog (*Eleutherodactylus johnstonei*) ten years after its introduction to Western French Guiana. Biol Invasions. 2011;13(8): 1767–77.

[52] Parris MJ, Cornelius TO. Fungal pathogen causes competitive and developmental stress in larval amphibian communities. Ecology. 2004;85(12): 3385–95.

[53] Retallick R, Miera V. Strain differences in the amphibian chytrid *Batrachochytrium dendrobatidis* and non-permanent, sub-lethal effects of infection. Dis Aquat Organ. 2007;75(3): 201–7. 10.3354/dao075201 17629114

[54] Borzée A, Kim JY, Cunha MAMd, Lee D, Sin E, Oh S, et al Temporal and spatial differentiation in microhabitat use: Implications for reproductive isolation and ecological niche specification. Integr Zool. 2016;11(5): 375–87. 10.1111/1749-4877.12200 27059098

[55] Lips KR, Reeve JD, Witters LR. Ecological traits predicting amphibian population declines in Central America. Conserv Biol. 2003;17(4): 1078–88.

[56] Brannelly LA, Hunter D, Skerratt LF, Scheele B, Lenger D, McFadden MS, et al Chytrid infection and post-release fitness in the reintroduction of an endangered alpine tree frog. Anim Conserv. 2015;19(2): 153–62.

[57] Cashins SD, Grogan LF, McFadden M, Hunter D, Harlow PS, Berger L, et al Prior infection does not improve survival against the amphibian disease chytridiomycosis. PloS one. 2013;8(2): e56747 10.1371/journal.pone.0056747 23451076 PMC3579874

[58] Richards-Zawacki CL. Thermoregulatory behaviour affects prevalence of chytrid fungal infection in a wild population of Panamanian golden frogs. P Roy Soc Lond B Bio. 2009;282(1819): rspb20091656. 10.1098/rspb.2009.1656 PMC2842693 19864287

[59] Weldon C, Du Preez LH, Hyatt AD, Muller R, Speare R. Origin of the amphibian chytrid fungus. Emerg Infect Dis. 2004;10(12): 2100 10.3201/eid1012.030804 15663845 PMC3323396

[60] Schloegel LM, Picco AM, Kilpatrick AM, Davies AJ, Hyatt AD, Daszak P. Magnitude of the US trade in amphibians and presence of *Batrachochytrium dendrobatidis* and ranavirus infection in imported North American bullfrogs (*Rana catesbeiana*). Biol Cons. 2009;142(7): 1420–6.

[61] Beebee TJC, Griffiths RA. The amphibian decline crisis: a watershed for conservation biology? Biol Cons. 2005;125(3): 271–85.

[62] Höbel G, Barta T. Adaptive plasticity in calling site selection in grey treefrogs (*Hyla versicolor*). Behav. 2014;151(6): 741–54.

[63] Ra N-Y, Sung H-C, Cheong S, Lee J-H, Eom J, Park D. Habitat use and home range of the endangered gold-spotted pond frog (*Rana chosenica*). Zool Sci. 2008;25(9): 894–903. 10.2108/zsj.25.894 19267598

[64] Kiesecker JM, Blaustein AR, Miller CL. Potential mechanisms underlying the displacement of native red-legged frogs by introduced bullfrogs. Ecology. 2001;82(7): 1964–70.

[65] Pryor GS. Tadpole nutritional ecology and digestive physiology: Implications for captive rearing of larval anurans. Zoo Biol. 2014;33(6): 502–7. 10.1002/zoo.21152 25182482

[66] Ficetola GF, Thuiller W, Miaud C. Prediction and validation of the potential global distribution of a problematic alien invasive species: the American bullfrog. Divers Distrib. 2007;13(4): 476–85.

