# Correction: Introduced bullfrogs are associated with increased *Batrachochytrium dendrobatidis* prevalence and reduced occurrence of Korean treefrogs

**DOI:** 10.1371/journal.pone.0190551

**Published:** 2017-12-28

**Authors:** Amaël Borzée, Tiffany A. Kosch, Miyeon Kim, Yikweon Jang

S1 Table and S2 Table are corrected for improved readability. Please view the corrected S1 Table and S2 Table below.

## Supporting information

S1 Table *Bd* prevalence for the two *Dryophytes* species. Sampling sites, sex of frogs and Bd prevalence for this study.
(DOCX)

S2 Table Call surveys for *Dryophytes suweonensis* and *Lithobates catesbeianus*. For “*L*. *catesbeianus*” and “*D*. *suweonensis*”, data is binary encoded: 0 = absent and 1 = present.
(DOCX)
