# Supplementary material for: Introduced bullfrogs are associated with increased Batrachochytrium dendrobatidis prevalence and reduced occurrence of Korean treefrogs
Source: PLoS One. 2017 May 31;12(5):e0177860. doi: 10.1371/journal.pone.0177860 (PMC5451047; doi:10.1371/journal.pone.0177860)
Supplement: S1 Table — Sampling sites, sex of frogs and Bd prevalence for this study. (DOCX) [file pone.0177860.s001.docx]

# Supporting information

**S1 Table. *Bd* prevalence for the two *Dryophytes* species.**

| Site | Ds M | Ds F | Dj M | Dj F | Prev Ds M | Prev Ds F | Prev Dj M | Prev Dj F |
| --- | --- | --- | --- | --- | --- | --- | --- | --- |
| 1 | 1 | 1 | 4 | 1 | 1 | 0 | 0 | 0 |
| 2 | 1 | 1 | 5 | 0 | 0 | 1 | 1 | 0 |
| 3 | 2 | 2 | 8 | 2 | 1 | 0 | 0 | 0 |
| 4 | 1 | 1 | 5 | 1 | 1 | 0 | 1 | 0 |
| 5 | 2 | 0 | 2 | 0 | 1 | 0 | 0 | 0 |
| 6 | 1 | 0 | 4 | 0 | 1 | 0 | 1 | 0 |
| 7 | 1 | 0 | 4 | 0 | 0 | 0 | 0 | 0 |
| 8 | 1 | 0 | 5 | 0 | 0 | 0 | 0 | 0 |
| 9 | 1 | 1 | 6 | 4 | 1 | 0 | 1 | 0 |
| 10 | 1 | 0 | 4 | 0 | 1 | 0 | 0 | 0 |
| 11 | 1 | 0 | 2 | 0 | 0 | 0 | 0 | 0 |
| 12 | 2 | 0 | 4 | 1 | 2 | 0 | 0 | 0 |
| 13 | 2 | 0 | 1 | 0 | 2 | 0 | 1 | 0 |
| 14 | 1 | 1 | 2 | 0 | 1 | 1 | 1 | 0 |
| 15 | 2 | 0 | 4 | 1 | 2 | 0 | 3 | 1 |
| 16 | 1 | 0 | 2 | 0 | 1 | 0 | 2 | 0 |
| 17 | 2 | 0 | 4 | 0 | 1 | 0 | 1 | 0 |
| 18 | 1 | 0 | 5 | 2 | 0 | 0 | 1 | 0 |
| 19 | 1 | 0 | 2 | 0 | 0 | 0 | 0 | 0 |
| 20 | 1 | 0 | 1 | 0 | 1 | 0 | 0 | 0 |
| 21 | 2 | 1 | 8 | 3 | 2 | 0 | 1 | 0 |
| 22 | 1 | 0 | 3 | 0 | 0 | 0 | 0 | 0 |
| 23 | 1 | 0 | 1 | 1 | 1 | 0 | 0 | 0 |
| 24 | 2 | 0 | 4 | 0 | 1 | 0 | 1 | 0 |
| 25 | 3 | 0 | 1 | 0 | 2 | 0 | 1 | 0 |
| 26 | 3 | 0 | 6 | 0 | 1 | 0 | 1 | 0 |
| 27 | 3 | 0 | 4 | 2 | 2 | 0 | 3 | 1 |
| 28 | 3 | 0 | 4 | 2 | 3 | 0 | 0 | 0 |
| 29 | 5 | 0 | 6 | 0 | 1 | 0 | 1 | 0 |
| 30 | 1 | 0 | 4 | 0 | 1 | 0 | 2 | 0 |
| 31 | 1 | 0 | 5 | 1 | 0 | 0 | 0 | 0 |
| 32 | 6 | 4 | 4 | 0 | 5 | 4 | 2 | 0 |
| 33 | 5 | 2 | 12 | 5 | 1 | 2 | 0 | 0 |
| 34 | 3 | 2 | 2 | 0 | 1 | 2 | 0 | 0 |
| 35 | 3 | 2 | 8 | 2 | 1 | 1 | 7 | 1 |
| 36 | 3 | 0 | 1 | 1 | 1 | 0 | 0 | 0 |
| 37 | 2 | 0 | 5 | 2 | 1 | 0 | 0 | 0 |
|  | | | | | | | | |

Ds is *Dryophytes suweonensis*, Dj is *D. japonicus* and Prev. stands for *Bd* prevalence*.* M for male and F for female.
