# Supplementary material for: Introduced bullfrogs are associated with increased Batrachochytrium dendrobatidis prevalence and reduced occurrence of Korean treefrogs
Source: PLoS One. 2017 May 31;12(5):e0177860. doi: 10.1371/journal.pone.0177860 (PMC5451047; doi:10.1371/journal.pone.0177860)
Supplement: S2 Table — For “L. catesbeianus” and “D. suweonensis”, data is binary encoded: 0 = absent and 1 = present. (DOCX) [file pone.0177860.s002.docx]

# Supporting information

**S2 Table. Call surveys for *Dryophytes suweonensis* and *Lithobates catesbeianus*.** For “*L. catesbeianus*” and “*D. suweonensis*”, data is binary encoded: 0 = absent and 1 = present.

| Number individuals *D. suweonensis* | Calling index *L. catesbeianus* | *L. catesbeianus* | *D. suweonensis* |
| --- | --- | --- | --- |
| 27 | 0 | 0 | 1 |
| 4 | 0 | 0 | 1 |
| 13 | 0 | 0 | 1 |
| 2 | 0 | 0 | 1 |
| 11 | 0 | 0 | 1 |
| 50 | 0 | 0 | 1 |
| 17 | 0 | 0 | 1 |
| 4 | 0 | 0 | 1 |
| 26 | 0 | 0 | 1 |
| 14 | 0 | 0 | 1 |
| 11 | 0 | 0 | 1 |
| 0 | 0 | 1 | 0 |
| 0 | 0 | 0 | 0 |
| 4 | 0 | 0 | 1 |
| 0 | 0 | 0 | 0 |
| 29 | 0 | 0 | 1 |
| 21 | 0 | 0 | 1 |
| 4 | 0 | 0 | 1 |
| 4 | 1 | 1 | 1 |
| 0 | 0 | 0 | 0 |
| 5 | 0 | 0 | 1 |
| 4 | 0 | 0 | 1 |
| 0 | 0 | 0 | 0 |
| 0 | 0 | 0 | 0 |
| 1 | 0 | 0 | 1 |
| 4 | 0 | 0 | 1 |
| 8 | 0 | 0 | 1 |
| 22 | 0 | 0 | 1 |
| 12 | 0 | 0 | 1 |
| 15 | 1 | 1 | 1 |
| 9 | 0 | 0 | 1 |
| 5 | 0 | 0 | 1 |
| 0 | 0 | 0 | 0 |
| 3 | 0 | 0 | 1 |
| 4 | 0 | 0 | 1 |
| 25 | 0 | 0 | 1 |
| 9 | 0 | 0 | 1 |
| 16 | 0 | 0 | 1 |
| 8 | 0 | 0 | 1 |
| 2 | 0 | 0 | 1 |
| 19 | 0 | 0 | 1 |
| 19 | 0 | 0 | 1 |
| 5 | 0 | 0 | 1 |
| 22 | 0 | 0 | 1 |
| 1 | 0 | 0 | 1 |
| 3 | 0 | 0 | 1 |
| 7 | 0 | 0 | 1 |
| 15 | 0 | 0 | 1 |
| 37 | 1 | 1 | 1 |
| 5 | 0 | 0 | 1 |
| 14 | 0 | 0 | 1 |
| 5 | 0 | 0 | 1 |
| 63 | 2 | 1 | 1 |
| 12 | 0 | 0 | 1 |
| 12 | 0 | 0 | 1 |
| 70 | 0 | 0 | 1 |
| 58 | 0 | 0 | 1 |
| 13 | 0 | 0 | 1 |
| 24 | 0 | 0 | 1 |
| 25 | 0 | 0 | 1 |
| 19 | 0 | 0 | 1 |
| 53 | 0 | 0 | 1 |
| 46 | 0 | 0 | 1 |
| 0 | 0 | 0 | 0 |
| 49 | 0 | 0 | 1 |
| 93 | 0 | 0 | 1 |
| 4 | 0 | 0 | 1 |
| 11 | 0 | 0 | 1 |
| 5 | 0 | 0 | 1 |
| 41 | 0 | 0 | 1 |
| 1 | 0 | 0 | 1 |
| 18 | 0 | 0 | 1 |
| 25 | 0 | 0 | 1 |
| 6 | 0 | 0 | 1 |
| 20 | 0 | 0 | 1 |
| 5 | 0 | 0 | 1 |
| 1 | 0 | 0 | 1 |
| 3 | 0 | 0 | 1 |
| 2 | 0 | 0 | 1 |
| 7 | 0 | 0 | 1 |
| 36 | 0 | 0 | 1 |
| 2 | 0 | 0 | 1 |
| 109 | 0 | 0 | 1 |
| 0 | 0 | 0 | 0 |
| 1 | 0 | 0 | 1 |
| 3 | 0 | 0 | 1 |
| 30 | 0 | 0 | 1 |
| 9 | 0 | 0 | 1 |
| 0 | 0 | 0 | 0 |
| 32 | 0 | 0 | 1 |
| 47 | 0 | 0 | 1 |
| 1 | 0 | 0 | 1 |
| 39 | 0 | 0 | 1 |
| 0 | 0 | 0 | 0 |
| 68 | 0 | 0 | 1 |
| 3 | 0 | 0 | 1 |
| 0 | 0 | 0 | 0 |
| 15 | 0 | 0 | 1 |
| 45 | 1 | 1 | 1 |
| 3 | 1 | 1 | 1 |
| 0 | 0 | 0 | 0 |
| 3 | 0 | 0 | 1 |
| 0 | 0 | 0 | 0 |
| 0 | 0 | 0 | 0 |
| 0 | 0 | 0 | 0 |
| 21 | 0 | 0 | 1 |
| 0 | 0 | 0 | 0 |
| 0 | 0 | 0 | 0 |
| 0 | 0 | 0 | 0 |
| 0 | 0 | 0 | 0 |
| 0 | 3 | 1 | 0 |
| 0 | 3 | 1 | 0 |
| 0 | 0 | 0 | 0 |
| 69 | 1 | 1 | 1 |
| 19 | 1 | 1 | 1 |
| 0 | 3 | 1 | 0 |
| 3 | 0 | 0 | 1 |
| 5 | 0 | 0 | 1 |
| 0 | 0 | 0 | 0 |
| 65 | 0 | 0 | 1 |
| 0 | 0 | 0 | 0 |
| 0 | 0 | 0 | 0 |
| 0 | 0 | 0 | 0 |
| 0 | 1 | 1 | 0 |
| 0 | 0 | 0 | 0 |
| 0 | 0 | 0 | 0 |
| 6 | 0 | 0 | 1 |
| 0 | 1 | 1 | 0 |
| 0 | 0 | 0 | 0 |
| 7 | 0 | 0 | 1 |
| 36 | 0 | 0 | 1 |
| 6 | 0 | 0 | 1 |
| 0 | 0 | 0 | 0 |
| 7 | 0 | 0 | 1 |
| 4 | 0 | 0 | 1 |
| 0 | 0 | 0 | 0 |
| 0 | 0 | 0 | 0 |
| 0 | 0 | 0 | 0 |
| 0 | 1 | 1 | 0 |
| 0 | 0 | 0 | 0 |
| 0 | 1 | 1 | 0 |
| 0 | 2 | 1 | 0 |
| 0 | 3 | 1 | 0 |
| 0 | 1 | 1 | 0 |
| 61 | 0 | 0 | 1 |
| 96 | 0 | 0 | 1 |
| 87 | 0 | 0 | 1 |
| 16 | 3 | 1 | 1 |
| 55 | 0 | 0 | 1 |
| 46 | 0 | 0 | 1 |
| 1 | 0 | 0 | 1 |
| 0 | 0 | 0 | 0 |
| 0 | 0 | 0 | 0 |
| 0 | 1 | 1 | 0 |
| 1 | 0 | 0 | 1 |
| 0 | 0 | 0 | 0 |
| 64 | 0 | 0 | 1 |
| 0 | 0 | 0 | 0 |
| 5 | 0 | 0 | 1 |
| 2 | 0 | 0 | 1 |
| 0 | 0 | 0 | 0 |
| 0 | 0 | 0 | 0 |
| 0 | 0 | 0 | 0 |
| 0 | 0 | 0 | 0 |
| 0 | 0 | 0 | 0 |
| 0 | 0 | 0 | 0 |
| 0 | 0 | 0 | 0 |
| 0 | 0 | 0 | 0 |
| 0 | 3 | 1 | 0 |
| 0 | 0 | 0 | 0 |
| 0 | 0 | 0 | 0 |
| 0 | 0 | 0 | 0 |
| 0 | 0 | 0 | 0 |
| 0 | 0 | 0 | 0 |
| 0 | 0 | 0 | 0 |
| 0 | 0 | 0 | 0 |
| 0 | 0 | 0 | 0 |
| 11 | 0 | 0 | 1 |
| 6 | 0 | 0 | 1 |
